# A pathogenic germline *BRCA2* variant in a patient with hypopharyngeal squamous cell carcinoma

**DOI:** 10.1002/ccr3.3548

**Published:** 2020-11-25

**Authors:** Tatiana Correa, Douglas E. Laux, Henry T. Hoffman

**Affiliations:** ^1^ Department of Otolaryngology – Head and Neck Surgery University of Iowa Iowa City IA USA; ^2^ Department of Internal Medicine–Hematology, Oncology & Bone Marrow Transplant University of Iowa Iowa City IA USA

**Keywords:** genetics, head and neck, molecular targeted therapies, oncology, pharmacology

## Abstract

Pathogenic germline *BRCA2* variants may be associated with an increased risk of hypopharyngeal squamous cell carcinoma that is more responsive to chemoradiation and chemotherapeutics targeting defective double‐strand DNA repair.

## Graphical abstract

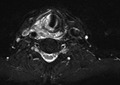

## 1 INTRODUCTION

Head and neck squamous cell carcinomas (HNSCCs) are prevalent and have high mortality rates that have remained largely unchanged in the last decade. In contrast, outcomes for many cancers have improved as a result of increased molecular understanding and personalized management. The persistent high mortality and clinical heterogeneity of HNSCC supports the need for a similar analysis.

Hypopharyngeal squamous cell carcinoma (SCC) has the poorest prognosis of all HNSCC subsites (oral cavity, oropharynx, larynx) thought in‐part due to detection at late stages. Over two‐thirds of patients with a hypopharyngeal SCC present with stage IV disease.^1^ Five‐year survival is much worse for stage IV (22%) compared to stage I (63%) and stage II (57%).^1^ Early detection of hypopharyngeal tumors is challenging due to the less accessible location and the nonspecific nature of initial symptoms, which often mimic common benign disorders such as laryngopharyngeal reflux.

Analysis of risk factors for cancer may direct more comprehensive evaluation for patients with early stage hypopharyngeal SCC. Known risk factors for hypopharyngeal SCC include tobacco use, heavy alcohol use, Plummer‐Vinson Syndrome‐associated iron deficiency anemia, and Fanconi Anemia.^1^, ^2^ To our knowledge, germline *BRCA2* variants have not been previously identified as a risk factor for hypopharyngeal SCC and awareness previously suggested associations with SCC at other head and neck subsites is limited. We present a case of hypopharyngeal SCC with a concurrent germline *BRCA2* variant and bring attention to potential diagnostic and management implications.

## 2 CASE REPORT

An 80‐year‐old healthy male patient with no history of tobacco or alcohol use presented to an otolaryngologist for throat pain, fever, right otalgia, and right maxillary sinus tenderness. He had previously been identified with a pathogenic germline *BRCA2* variant during successful management of intraductal breast carcinoma, pancreatic adenocarcinoma, and prostatic adenocarcinoma over 10 years prior. He otherwise had a maternal history of breast cancer during the 8th and 9th decade and two healthy adult children with germline BRCA2 variant mutations. At the initial otolaryngology visit, physical examination was normal, and he was empirically treated for bacterial sinusitis.

**Figure 1:**
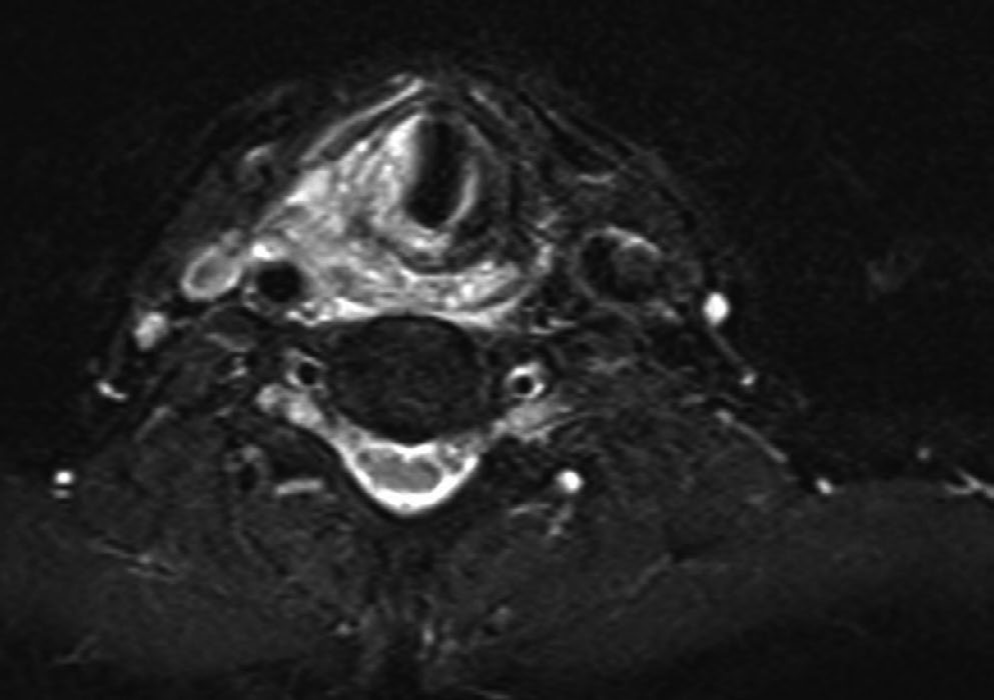
T2 weighted axial MRI image of tumor invading right thyroid cartilage and encasing right carotid

On follow‐up, the patient had improvement of initial symptoms but had developed a nonproductive cough. His examination at that time was remarkable for a small pharyngeal ulcer, and antigastroesophageal reflux therapy was started. Subsequently, the patient underwent CT larynx without contrast by his Oncologist for additional workup of fatigue in the context of ongoing otalgia, sinus tenderness, and cough. This imaging revealed an asymmetric soft tissue prominence at the larynx with thickening along the adjacent right hypopharynx, a 1.3 cm lymph node and fat stranding at the right carotid space. Laryngopharyngitis was diagnosed based on repeat examination and review of imaging. A primary care physician then obtained a noncontrast MRI for new headaches incidentally identifying a transglottic mass invading thyroid cartilage with encasement of the right carotid, and a 1.6 cm right level IIA lymph node (Figure 1). The patient was referred to our tertiary care center for concern of hypopharyngeal malignancy.

**Figure 2:**
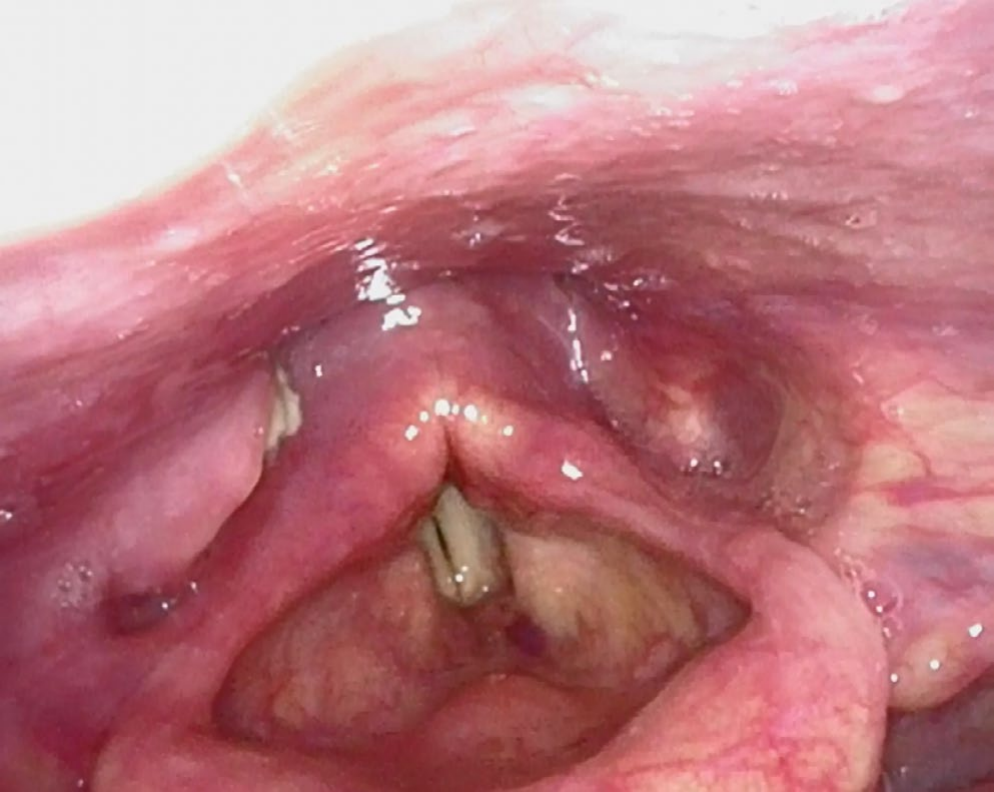
Right piriform sinus mass extending to right pharyngeal wall with minimal medial disruption in appearance of glottic and supraglottic structures

Evaluation with flexible fiberoptic laryngoscopy revealed a mass at the right pharyngeal wall extending to the right pyriform sinus (Figure 2). Ultrasound‐guided needle biopsy was attempted twice revealing acute inflammation and keratinized squamous cell epithelium of the right piriform sinus, respectively. Biopsy of the right level II lymph node showed chronic lymphocytic leukemia. A biopsy of the right piriform sinus under general anesthesia was then completed revealing well‐differentiated keratinizing squamous cell carcinoma.

A diagnostic PET/CT showed an FDG‐avid soft tissue mass centered on the right piriform sinus with extension into the right parapharyngeal soft tissues, thyroid cartilage, and prevertebral space to the level of the cricoid cartilage. Chemoradiation (using weekly carboplatin and paclitaxel) was successfully completed for the stage IVa (cT4b, cN0, M0) disease. A twelve‐week postchemoradiation PET/CT revealed complete resolution of cancer, and the patient remained disease‐free at most recent 3‐year follow‐up.

## 3 DISCUSSION

Hypopharyngeal SCCs account for <10% of HNSCCs and are difficult to visualize at early stages.^1^ Patients diagnosed with hypopharyngeal SCCs are predominantly males in their early 60 seconds with a history of smoking and heavy alcohol use. These risk factors are a key determinant of clinical suspicion since tumors are associated with nonspecific early symptoms.

*BRCA2* variants in HNSCC have been reported in the context of genetic variants in some patients with Fanconi Anemia, which is associated with hypopharyngeal SCC. These patients are typically young adult females with history of hematologic malignancies who develop HNSCCs in their third and fourth decade.^2^ One other truncating *BRCA2* variant has been linked to hypopharyngeal SCC; however, lung SCC is the most common with this variant and breast cancer is rare.^3^ In some studies of families with a *BRCA2* variant mutations, a significant increased risk of laryngeal and pharyngeal cancer has been identified.^3^

The exact *BRCA2* variant mutation present in our patient is unknown; however, there are notable findings in his cancer history suggesting he may carry a less‐common variant associated with cancer at later age and sites not traditionally associated with *BRCA2* mutations. Our patient had no malignancy until his 60 seconds when he developed intraductal breast carcinoma. He then developed stage III pancreatic adenocarcinoma, which was resected and then treated by oxaliplatin chemoradiation for local recurrence. His third malignancy, stage I prostatic adenocarcinoma, occurred in his late 60s and he had remained cancer‐free until presenting with hypopharyngeal SCC. Our patient’s family history did not reveal history cancer until advanced age and two middle aged adult children who also had a germline *BRCA2* variant.

*BRCA2* variants can result in many cancer phenotypes due to the complex and vital role of the BRCA2 protein in DNA homologous recombination (HR) repair. The *BRCA2* gene is involved in the Fanconi Anemia (FA)/BRCA pathway for double‐strand DNA break repair. Variants involving the FA/BRCA pathway have been implicated in tumor development for both sporadic and Fanconi Anemia‐related HNSCC.^3^, ^4^ In this pathway, BRCA2 and other FA/BRCA proteins form a complex for repair of DNA cross‐links through HR. Pathologic variants in any of the genes involved in the HR repair complex can impair DNA repair and potentiate cancer formation.

In addition to a potential role in hypopharyngeal SCC development, *BRCA2* variants may have management implications by enhancing platinum‐based chemotherapy response in cancer cells as a result of deficient DNA repair leading to increased platinum‐DNA adducts.^4^ This mechanism has been observed in HNSCCs *in* *vitro* ^4^ and is consistent with the excellent response to chemoradiation observed in our patient. In other malignancies including ovarian, prostate, and pancreatic cancers, pathogenic germline *BRCA2* variants have been associated with clinically distinct tumor phenotypes that respond better to agents targeting the FA/BRCA HR pathway.^5^ Notably, our patient demonstrated excellent response to platinum‐based chemotherapy while undergoing treatment of locally recurrent pancreatic adenocarcinoma. He also developed prostate cancer recurrence at the time of his hypopharyngeal SCC diagnosis that was refractory to chemical castration and ultimately controlled with a PARP inhibitor, which also targets the FA/BRCA HR pathway.

## 4 CONCLUSION

This case report draws attention to a potential link between pathogenic germline *BRCA2* variants and hypopharyngeal cancer. Patients with pathogenic germline *BRCA2* variants may benefit from more intensive workup when exhibiting symptoms commonly attributed to laryngopharyngitis. Hypopharyngeal SCC associated with *BRCA2* variants and potentially other associated HNSCCs may show increased chemo‐radio‐sensitivity. Genetic screening to direct use of platinum‐based therapies, immunotherapy, and PARP inhibitors for other *BRCA2*‐related cancers is currently underway suggesting similar targeted therapy potential in HNSCC.

## CONFLICT OF INTEREST

No funding or conflicts of interest to disclose for Dr Correa. No funding or conflicts of interest to disclose for Dr. Laux. Dr. Hoffman is an author for UpToDate and a research consultant for OtoMotion Inc.

## AUTHOR CONTRIBUTIONS

TC wrote the manuscript, conducted literature review and synthesized correlations with Fanconi Anemia and targeted cancer therapies. DEL provided oversight of oncologic interpretations and critically revised the manuscript. HTH contributed the presented case, design of the manuscript and critically revised the manuscript. All authors reviewed and approved the final version of the manuscript.

## ETHICS STATEMENT

Written informed consent for publication of the case and images was obtained from the patient prior to the writing of this case report.

## Data Availability

No dataset was generated or analyzed for this publication. Clinical data to support the findings presented in this case report are included within the article.

## References

[1] Eckel HE , Bradley PJ . Natural History of Treated and Untreated Hypopharyngeal Cancer. Adv Otorhinolaryngol. 2019;27–34. 10.1159/000492305 30943503

[2] Chandrasekharappa SC , Chinn SB , Donovan FX , et al. Assessing the spectrum of germline variation in Fanconi anemia genes among patients with head and neck carcinoma before age 50. Cancer. 2017;123(20):3943–3954. 28678401 10.1002/cncr.30802 PMC5853120

[3] van Asperen CJ , Brohet RM , Meijers‐Heijboer EJ , et al. Cancer risks in BRCA2 families: estimates for sites other than breast and ovary. J Med Genet. 2005;42(9):711–719. 16141007 10.1136/jmg.2004.028829 PMC1736136

[4] Kemp SRM‐D , Brink A , Meulen IHVD , et al. The FA/BRCA pathway identified as the major predictor of cisplatin response in head and neck cancer by functional genomics. Mol Cancer Ther. 2016;16(3):540–550. 27980104 10.1158/1535-7163.MCT-16-0457

[5] Wattenberg MM , Asch D , Yu S , et al. Platinum response characteristics of patients with pancreatic ductal adenocarcinoma and a germline *BRCA1*, *BRCA2* or *PALB2* mutation. Br J Cancer. 2020;122:333–339. 31787751 10.1038/s41416-019-0582-7 PMC7000723

